# Profiling of Circulating MicroRNAs after a Bout of Acute Resistance Exercise in Humans

**DOI:** 10.1371/journal.pone.0070823

**Published:** 2013-07-29

**Authors:** Shuji Sawada, Michihiro Kon, Shogo Wada, Takashi Ushida, Katsuhiko Suzuki, Takayuki Akimoto

**Affiliations:** 1 Faculty of Sport Sciences, Waseda University, Cooperative Major in Advanced Health Science, Tokyo University of Agriculture and Technology/Waseda University, Tokorozawa, Saitama, Japan; 2 Department of Sports Sciences, Japan Institute of Sports Sciences, Nishigaoka, Tokyo, Japan; 3 Division of Regenerative Medical Engineering, Center for Disease Biology and Integrative Medicine, Graduate School of Medicine, The University of Tokyo, Hongo, Tokyo, Japan; UT MD Anderson Cancer Center, United States of America

## Abstract

Recent studies have revealed a new aspect of physiological regulation in which microRNAs (miRNAs) play fundamental roles in diverse biological and pathological processes. Furthermore, it was recently discovered that miRNAs are stably secreted into blood and that circulating miRNAs may play important roles in cell–cell communication. Here, we examined whether the circulating miRNA profile is affected by acute resistance exercise. Twelve males performed a resistance exercise session (bench press and leg press), consisting of five sets of 10 repetitions at 70% of maximum strength, with a 1 min rest between sets. Blood samples were taken before exercise, and at 0 and 60 min, 1 day, and 3 days after exercise. The circulating miRNA profile was determined by microarray analysis. Quantitative real-time PCR confirmed that the miR-149* level increased three days after resistance exercise. In contrast, the miR-146a and miR-221 levels decreased three days after resistance exercise. Our findings suggest that circulating miRNA levels change in response to acute resistance exercise, and miRNAs may play important roles in resistance-exercise-induced adaptation.

## Introduction

Recent studies have revealed a new aspect of physiological regulation in which small, non-coding RNAs, known as microRNAs (miRNAs), play fundamental roles in diverse biological and pathological processes, including differentiation and morphogenesis [1]. miRNAs are approximately 22 nucleotides long and inhibit the expression of the target mRNAs through translational repression or mRNA decay by interacting with the 3′ untranslated regions (UTRs) of target mRNAs [2], [3]. A single miRNA can regulate multiple mRNAs and can alter the levels of critical regulators. It was estimated that miRNAs regulate nearly two-thirds of the mammalian genome [4]. Thus, miRNAs play fundamental roles in diverse biological and pathological processes [5], [6].

It was recently discovered that miRNAs are stably secreted into and circulate via the bloodstream [7]. The circulating miRNAs may be protected from degradation in blood by several complementary mechanisms, including their incorporation into phospholipid bilayer-encapsulated vesicles, the “exosome” [8], or by the formation of RNA-binding protein complexes [9]. The discovery of stable circulating miRNAs raises questions over the function of these miRNAs. In fact, recent studies suggested that miRNAs are involved in cell–cell communication [10], [11], [12] and that circulating miRNAs, like hormones and cytokines, mediate gene expression in the target cells.

Resistance exercise is a potent stimulator of muscle protein synthesis and muscle cell growth. The exercise-induced increase in protein synthesis is associated with morphological and functional adaptations of skeletal muscle [13], [14] via the induction of hormones and cytokines, including testosterone, dehydroepiandrosterone, and insulin-like growth factor-1 (IGF-1) [15], [16], [17]. In fact, acute resistance exercise increases some of these hormones. It appears that this acute response is more critical to muscle adaptation than are chronic changes in resting hormonal concentrations [18]. However, it has not been determined whether resistance exercise affects circulating miRNAs, even though circulating miRNAs have been extensively studied as biomarkers of various diseases, such as cancer [19]. The identification of circulating miRNAs that are specifically regulated by exercise could reveal unique biomarkers of exercise physiology and would provide further insight into the molecular control of exercise adaptations [20]. Therefore, we performed a comprehensive microarray analysis of circulating miRNAs and their responses to acute resistance exercise. We then performed quantitative real-time reverse-transcriptase polymerase chain reaction (RT-PCR) to quantify the expression of specific resistance-exercise-induced miRNAs.

## Methods

### Ethical approval and study participants

Twelve healthy nonsmoking males participated in this study. The physical characteristics of the subjects are shown in Table 1. All of the subjects were physically active and had experience of recreational exercise training. None of the subjects was participating in a regular training program at the start of the study. The subjects reported that they did not use any medications (e.g., anabolic steroids and sympathoadrenal drugs) during the experimental trials. Written informed consent was obtained from all of the subjects. Ethical approval for this study conformed to the standards of the Declaration of Helsinki and the protocol was approved by the Japan Institute of Sports Sciences Ethics Committee.

**Table 1:** Subject characteristics.

Height (cm)	172.4±1.2
Body mass (kg)	68.9±2.0
BMI (kg/m^2^)	23.2±1.2
Age (years)	29.9±1.2

BMI, body mass index. Data are presented as means ± SE.

### Exercise trials

The subjects visited the laboratory 1–2 weeks before the experimental trials. The one repetition maximum (1 RM) for the bench press and bilateral leg press exercises were measured using weight-stack machines. Before assessing the 1 RM, the subjects were invited to stretch for several minutes; they then performed two warm-up sets of ten repetitions. The load was increased until the subjects were unable to lift the load. The subjects rested for ≥3 min between each trial to avoid fatigue. Three to five trials were conducted to determine 1 RM.

The resistance exercise consisted of two consecutive exercises (bench press and bilateral leg press), consisting of five sets of 10 repetitions at 70% of 1 RM. The subjects were allowed to rest for 1 min between each set and exercise. The subjects were instructed to lift and lower the load at a constant velocity, taking about 2 s for each repetition. If the load became too heavy, the subject was assisted. The range of motion in each exercise was from 90° to 0° (0° at full extension). The trials were performed between 08:00 and 11:30 a.m. to avoid diurnal variations in metabolic and hormonal responses.

### Blood sampling and biochemical analysis

After an overnight fast, the subjects attended the laboratory and rested for 30 min before the first blood collection. Venous blood samples were obtained from the subject’s forearm before (Pre), and at 0 min (immediately after exercise; Post 0 min), 60 min (Post 1 h), 1 day (Post 1d), and 3 days (Post 3d) after exercise. Serum and plasma samples were prepared by centrifuging the samples at 3000 rpm for 15 min, and were stored at –80°C until analysis. Creatine kinase (CK) activity was measured by the UV (NAC) method, as recommended by GSCC [21], and C-reactive protein (CRP) concentrations were measured by nephelometry. Blood samples were also obtained from the fingertip to measure lactate concentrations using an automatic lactate analyzer (Lactate Pro; ARKRAY, Kyoto, Japan).

### Assessment of subjective fatigue during exercise trial

A visual analogue scale was used to assess fatigue subjectively. The subjects were asked to rate their degree of fatigue and tension by marking a 100 mm line with a pen at some point between 0 on the left end (no fatigue) and 100 on the right end (maximum fatigue). The visual analogue score was recorded at the time of blood sampling. The heart rates and subjective fatigue of the subjects are shown in Table 2.

**Table 2:** Changes in heart rate and subjective fatigue during the exercise trial.

	Pre	Post 0h	Post 1h	Post 1d	Post 3d
Heart rate (beat/min)	67.4±67.4	92.2±4.7	67.2±3.2	ND	ND
Subjective fatigue (0–100)	5.6±2.2	66±7.7	16.1±3.3	18.4±4.0	7.1±2.3

Subjective fatigue was assessed on a visual analogue scale. ND: not determined. Data are presented as means ± SE (n = 12).

### RNA isolation

RNA was isolated from serum samples as previously described [19], with some modifications. Briefly, 400 µl of human serum was thawed and mixed with five volumes of QIAzol reagent and vortexed for 10 s. The samples were then incubated at room temperature for 5 min to inactivate RNases. To allow for normalization of sample-to-sample variation in RNA isolation, synthetic *C. elegans* miRNAs cel-miR-39, cel-miR-54, and cel-miR-238 (synthetic RNA oligonucleotides synthesized by Fasmac, Odawara, Japan) were added (25 fmol of each oligonucleotide in a 5 µl total volume) to each sample. The samples were then stored at –80°C until use. After thawing the samples, one volume of chloroform was added. We then followed the manufacturer’s protocol, and the entire aqueous phase from each sample was loaded onto a single affinity column. RNA was eluted with 50 µl of elution solution.

### miRNA profiling

For microarray expression profiling of miRNAs, total RNA from serum samples were labeled and hybridized to a miRCURY™ LNA miRNA Array (Exiqon, Vedbaek, Denmark) in accordance with the manufacturer’s instructions. The miRCURY™ LNA miRNA Array contains control probes, mismatch probes, and capture probes, which match all the miRNAs in human, mouse and rats, as annotated in miRBase Release 17.0. The control probes include 10 spike-in control probes to ensure optimal labeling and hybridization, as well as 8 negative capture probes and 12 capture probes that hybridize small nuclear RNAs. RNA labeling and hybridization was completed according to the manufacturer’s instructions. Briefly, the RNAs from each sample were labeled with Hy3 using the miRCURY™ LNA miRNA Array labeling kit. After labeling, the samples were loaded onto the microarray slide and incubated for 16–18 h at 60°C. After hybridization, the slides were washed, dried by centrifugation, and scanned using an Agilent Microarray Scanner (model G2565B). The mean values of the replicate spots of each miRNA on the microarray were normalized using a global normalization procedure. The correction factor was calculated by dividing the sum intensity of each sample by the mean intensity of all samples. The normalized values were calculated by multiplying the mean intensity of each miRNA by the correction factor. The expression levels for each serum sample were then computed relative to the expression level observed in the universal control.

The Hy5:Hy3 ratio (universal control/sample) was first converted to a log_2_ value. The gene expression signatures in response to a bout of resistance exercise were then evaluated using the following selection criteria; fold-change >1.5, coefficient of variation <50%, and high Hy3 signal >1.5× the median signal. We chose targets that met the fold-change criterion in at least one sample. The target was then removed if multiple samples did not meet the criteria or if the validation tendency was not matched.

### miRNA quantification

The TaqMan MicroRNA Reverse Transcription Kit and TaqMan MicroRNA assays (Applied Biosystems, Foster City, CA) were used for real-time PCR quantification of mature miRNA expression [22]. To quantify miRNAs, each RT reaction contained 10 ng of purified total RNA. The reactions were incubated for 30 min at 16°C, 30 min at 42°C, and 5 min at 85°C. Real-time PCR reactions for each miRNA (10 µl reaction volume) were performed in triplicate, and each 10 µl reaction mixture included 0.67 µl of the RT product. Reactions were performed using an Applied Biosystems 7500 Fast Real-Time PCR system in 96 well plates at 95°C for 10 min, followed by 40 cycles of 95°C for 15 s and 60°C for 1 min.

The target miRNAs were selected in two ways: we first selected four miRNAs based on the microarray analysis, as described above. We also selected the following eight miRNAs that were validated in an earlier study: miR-20a, miR-21, miR-133a, miR-146a, miR-210, miR-221, miR-222, and miR-328 [20].

The results of real-time PCR were first presented as Ct values, so we calculated the ΔΔCt value for each miRNA using the Ct value of cel-miR-39 and the Ct value at Pre. Using this, we calculated the fold-change for times Post 0 min, Post 1h, Post 1 d, and Post 3 d, relative to Pre. We evaluated miR-16, a stable and abundant miRNA in blood, and confirmed the accuracy of miRNA isolation and quantification of circulating miRNAs.

### Statistical analysis

Before statistical analysis, all data, except hemoglobin values, were corrected for changes in serum volume using the method of Costill and Fink [23], [24]. All data were analyzed by one-way analysis of variance with repeated measures. If significant differences existed, *post hoc* analyses (Bonferroni multiple comparison test) were performed. We also used Spearman’s rank correlation coefficient to analyze the changes in miRNA levels and resistance-exercise-related blood parameters. The level of statistical significance was set at *P*<0.05. Data are expressed as means ± standard error (SE).

## Results

### Global identification of circulating miRNAs in response to resistance exercise

**Figure 1:**
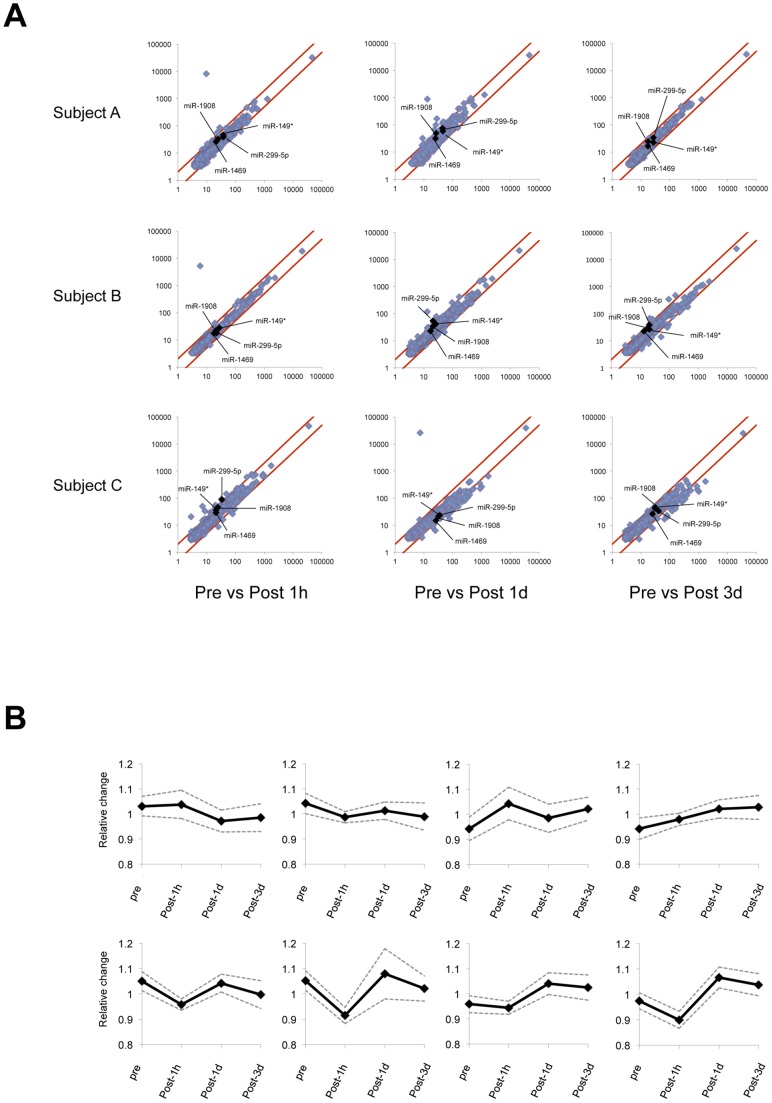
Microarray analysis of circulating miRNAs following acute resistance exercise. (A) Accuracy of hybridization of miRNA microarray. The levels of very few miRNAs changed by >1.5-fold change at any time. (B) Self-organization map clusters generated using the 843 regulated expression profiles. We specified eight clusters and an algorithm grouped them into discrete clusters. Expression profiles were determined at before, 60 min after (Post 1h), one day after (Post 1d), and three days after exercise (Post 3d), and are designated by black dots from left to right, where Pre is set as 0 in the log_2_ ratio between control samples. The thick and thin lines represent the mean expression level and standard deviation, respectively.

We randomly selected three individuals (12 samples; three individuals × four time points) to determine their miRNA expression profiles. Pooled total RNA from the 12 samples was used as a universal reference. The expression profiles were determined using miRCURY™ LNA miRNA Arrays (GEO accession numbers: GSE48405, Gene Expression Omnibus). LNAs are a class of conformationally restricted nucleotide analogues that increase the affinity of an oligonucleotide for its complementary RNA or DNA target. The Exiqon miRNA array is one of the most comprehensive probe sets available on an array platform [25], and allows for in-depth analysis of miRNA expression (Fig. 1A). After RNA hybridization and array analysis, K-means clustering and self-organization map [26] were used to analyze and assemble the data. These methods group the miRNAs with similar expression patterns. Eight cluster groups were used in this study (Fig. 1B). Based on the criteria described in the Method (fold-change >1.5, coefficient of variation <50%, and high Hy3 signal >1.5× the median signal), only four miRNAs (miR-149*, miR-299-5p, miR-1469 and miR-1908) were selected.

### Quantification of changes in circulating miRNA levels in response to resistance exercise

We then attempted to determine the changes in the four miRNAs identified from the microarray data and eight miRNAs that were reported to change in response to endurance exercise [20]. Among these miRNAs, we could not measure miR-1469 because the TaqMan MicroRNA assays for miR-1469 was not available.

**Figure 2:**
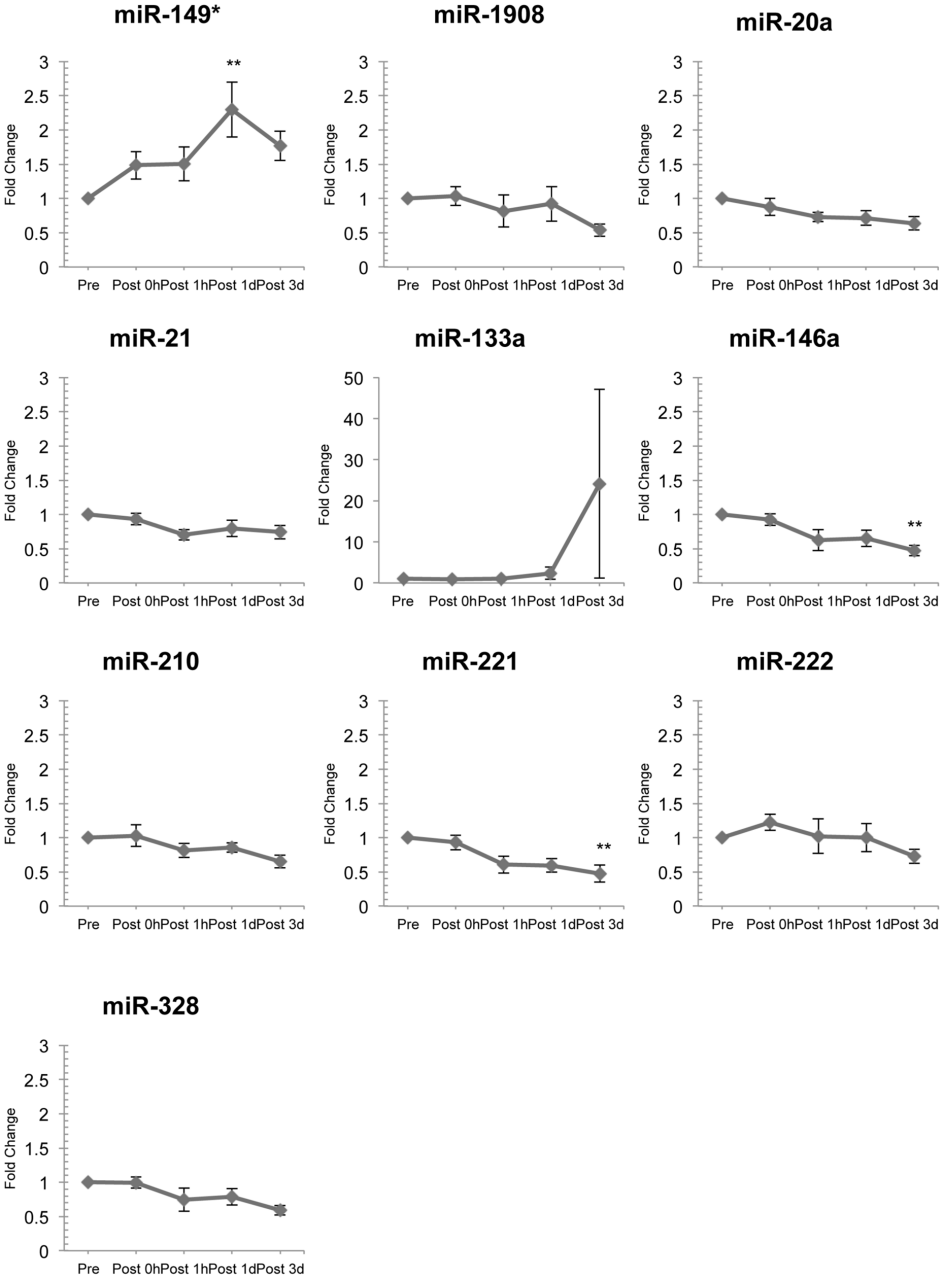
Changes in circulating miRNA levels following acute resistance exercise. The miR-149* level had increased significantly three days after resistance exercise. miR-146a and miR-221 levels had decreased significantly five days after resistance exercise. Levels of the other miRNAs did not change significantly. *P<0.05, **P<0.01.

The quantitative analysis showed that the expression levels of miR-149* were significantly increased at Post 3d of resistance exercise (Fig. 2). The analysis also showed that the expression levels of miR-146a and miR-221 were significantly reduced at Post 3d of exercise (Fig. 2). Unfortunately, we could not detected miR-299-5p, possibly because only a small amount was expressed. The other miRNAs (miR-1908, miR-20a, miR-21, miR-133a, miR-210, miR-222, and miR-328) did not change significantly following resistance exercise (Fig. 2). The Ct values for the miRNAs were approximately 28 (miR-21), 29 (miR-20a), 30 (miR-146a, miR-221), 31 (miR-222), 33 (miR-149*, miR-210, miR-328, miR-1908), and 34 (miR-133a).

### Correlations between miRNA levels and resistance-exercise-related blood parameters

**Figure 3:**
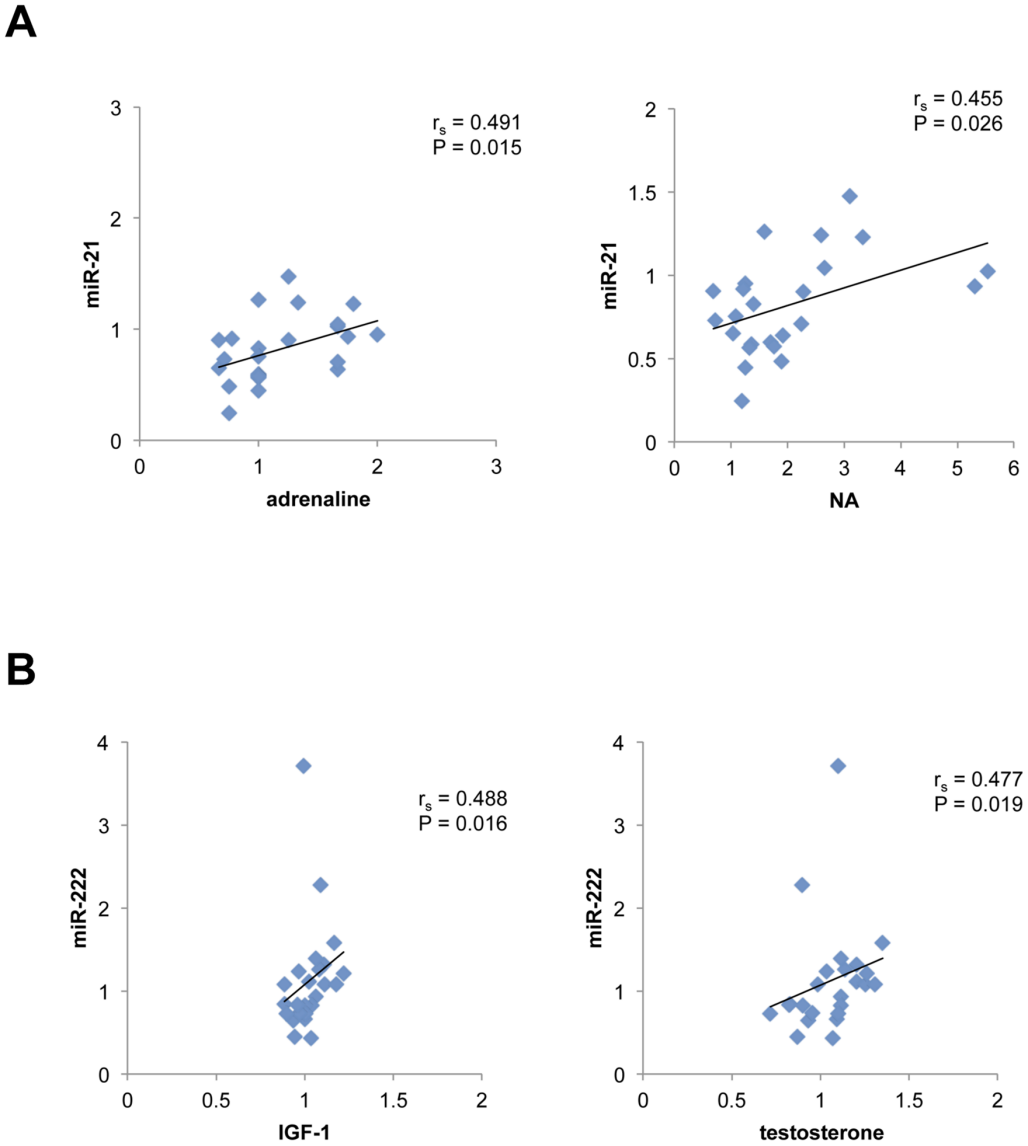
Correlations between circulating miRNA levels and resistance-exercise-related blood parameters following acute resistance exercise. (A) Adrenaline and noradrenaline levels correlated weakly but significantly with miR-21 levels, yielding correlation coefficients of 0.491 and 0.455, respectively; (B) miR-222 levels correlated weakly with IGF-1 and free testosterone levels, yielding correlation coefficients of 0.488 and 0.477, respectively. NA, noradrenaline.

We analyzed the correlations between changes in miRNA levels and resistance-exercise-related blood parameters, including growth hormone (GH), IGF-1, cortisol, adrenaline, noradrenaline, testosterone, CK activity, myoglobin, and interleukin-6 (IL-6) (Table 3). We found some significant but weak correlations between the changes in plasma catecholamine levels and miR-21 (Fig. 3A). Furthermore, IGF-1 and free testosterone levels correlated weakly with miR-222 levels (Fig. 3B).

**Table 3:** Correlations between changes in miRNAs and resistance-exercise-related blood parameters.

	GH	IGF-1	Cortisol	Adrenaline	NA	Testosterone	CK	Mb	IL-6
miR-149*	–0.268	–0.015	0.068	0.084	0.158	–0.145	0.010	–0.160	0.229
miR-1908	–0.373	0.137	0.029	0.086	0.003	–0.009	–0.057	–0.194	0.055
miR-20a	–0.141	0.220	0.221	0.320	0.389	–0.021	0.217	0.040	0.216
miR-21	–0.037	0.357	0.250	0.491*	0.455*	0.052	0.228	0.061	–0.013
miR-133a	–0.246	–0.096	0.245	0.180	0.143	–0.300	0.186	0.166	0.143
miR-146a	–0.154	0.336	0.162	0.222	0.270	0.208	0.084	–0.096	–0.136
miR-210	–0.303	0.284	0.107	0.296	0.183	0.128	0.096	–0.060	–0.092
miR-221	–0.329	0.213	0.101	0.256	0.136	0.042	0.037	–0.167	–0.128
miR-222	–0.090	0.487*	0.159	0.311	0.293	0.477*	0.191	0.036	–0.133
miR-328	–0.214	0.282	0.080	0.246	0.223	0.217	0.118	–0.207	–0.068

GH, growth hormone; IGF-1, insulin-like growth factor 1; NA, noradrenaline; CK, creatine kinase; Mb, myoglobin; IL-6, interleukin-6. *: p<0.05.

## Discussion

In the present study, we confirmed that some circulating miRNAs were influenced by acute resistance exercise. These results may imply that circulating miRNA could be used as possible biomarkers of exercise physiology, and provide novel insight into the regulatory roles of circulating miRNAs in physiological adaptations to resistance exercise.

Several studies have determined the impact of acute exercise on circulating miRNAs, and some studies have reported the effects of exercise on miRNAs in skeletal muscle in humans [27], [28]. In fact, one study evaluated the effects of endurance exercise on circulating miRNAs. For example, Baggish et al. (2011) reported that the circulating levels of the miRNAs miR-146a, miR-21, miR-221, and miR-222 increased significantly after a bout of acute aerobic exercise [20]. Although that study provided the first evidence that acute endurance exercise affected the circulating levels of miRNA, there were some limitations. In particular, they did not correct for changes in plasma volume to exclude the effect of endurance-exercise-induced hemoconcentration [23], [24]. Uhlemann et al. (2012) determined changes in 2 miRNAs and reported that miR-126 levels increased after acute endurance exercise, miR-133 levels increased after acute resistance exercise, and levels of both miR-126 and miR-133 increased after a marathon [29].

We initially hypothesized that some circulating miRNAs, particularly muscle-specific miRNAs (e.g., miR-1 and miR-133), were highly responsive to a bout of resistance exercise because of muscle recruitment during resistance exercise. Unexpectedly, however, the microarray analysis revealed no changes in circulating levels of muscle-specific miRNAs following acute resistance exercise. These results are consistent with those of an earlier study of acute endurance exercise [20]. These results suggest that tissue-specific miRNAs are not secreted into blood by passive transport; instead, some miRNAs may be selectively released to exert their roles in other tissues or cells.

Until now, no systematic study has evaluated the circulating miRNA profile after acute resistance exercise. We, for the first time, examined the effects of acute resistance exercise on the miRNA profile by comprehensive microarray analysis and performed quantitative analysis. To achieve this, we first performed a comprehensive, high-throughput screening of thousands of miRNAs using an LNA-based microarray, focusing on three subjects at four time points, rather than examining a large sample. To confirm the profiles using a more reliable quantitative method, we used TaqMan real-time RT-PCR assays to examine the miRNA profiles in 12 subjects at five time points. These analyses showed that the circulating levels of miR-149* increased, whereas those of miR-146a and miR-221 decreased significantly after acute resistance exercise. To our surprise, only three of the 1458 miRNAs were affected by acute resistance exercise. The specific changes in these circulating miRNAs suggest that they play some roles in resistance-exercise-induced muscle adaptation. However, we did not elucidate the physiological significance of the changes in circulating miRNAs.

Some studies have revealed changes in circulating miRNAs in pathological conditions. For example, serum miR-146a levels were significantly decreased in septic patients [30] and in patients with systemic lupus erythematosus [31], but were increased in patients with liver cirrhosis or hepatocellular carcinoma [32], and in patients infected with hepatitis C virus [33]. In addition, it was recently reported that the circulating levels of miR-146a were significantly elevated in patients with acute coronary syndrome [34]. Meanwhile, circulating miR-221 levels were significantly increased in patients with pancreatic cancer [35], colorectal cancer [36], gastric cancer [37], and hepatocellular carcinoma [38]. Consequently, these miRNAs might be related to the pathogenesis of these diseases. In contrast, no report has documented changes in circulating miR-149*. Further studies should focus on the physiological meaning of this increase in circulating miR-149*.

We found that the circulating miR-146a level had decreased three days after acute resistance exercise, but it is reported that acute aerobic exercise increased circulating miR-146a levels in an earlier study [20]. In contrast, we found that the miR-21 and miR-222 levels did not change after a bout of resistance exercise, although these miRNAs have been reported to increase after a bout of endurance exercise [20]. These differences suggest that the changes in circulating miRNAs may be influenced by the types of exercise. Therefore, further systematic studies should be conducted to investigate the effects of exercise type, duration, and intensity on the circulating miRNA profile.

To determine the relationship between changes in miRNA levels and those in resistance-exercise-induced blood parameters, we performed correlation analysis and found some significant but weak correlations between the changes in several circulating miRNAs and the changes in plasma catecholamines (Fig. 3A), IGF-1, and free testosterone (Fig. 3B). These results suggest that these hormones may be associated with the circulating miRNAs. Therefore, future studies should determine whether these miRNAs were generated in skeletal muscle or in other tissues after resistance exercises, and therefore clarify the effects of acute resistance exercise on the biogenesis of circulating miRNAs. It is also necessary to study the function of these circulating miRNAs in relation to muscle hypertrophy.

Circulating miRNAs are easily sampled in humans using blood samples, and stable following freezing, thawing, and temperature changes [19]. This explains why circulating miRNA levels are frequently examined in epidemiologic studies. However, a standardized detection method of circulating miRNAs has not been established.

Finally, the inconsistency between the results of the comprehensive analysis and the quantitative analysis has to be discussed. We chose four miRNAs based on the changes observed in the LNA-based microarray, and quantitative real-time RT-qPCR confirmed the changes in miR-149* following resistance exercise. However, we could not determine the changes in miR-1469 because no TaqMan miRNA assay kit was available; nor miR-299-5p, possibly because it was only expressed at low levels. We failed to confirm the changes in miR-1908 following resistance exercise. One possible explanation for this inconsistency may be the individual variations of the miRNAs because we subjected 12 serum samples (three individuals × four time points) to microarray analysis, whereas we measured miRNA expression in 60 samples (12 individuals × five time points) by real-time RT-qPCR.

Another possibility may be the difference in detection methods used. Three principal methods are currently used to measure the levels of miRNA expression: real-time RT-PCR, microarray hybridization, and massively parallel/next-generation sequencing. Their relative performances are still being evaluated [39]. In this study, the LNA-based microarray was used for the comprehensive analysis, while TaqMan DNA probe-based real-time PCR assays were used for quantitative analysis. Real-time RT-PCR is a well-accepted technique in the field and is often used to validate the results of microarray and sequencing analyses, although microarrays are still the best choice as a standardized genome-wide assay that is amenable to high-throughput applications [40]. It has been reported that each of these three platforms performs similarly in terms of intraplatform reproducibility and the reproducibility of data within one platform, whereas the interplatform reproducibility of different platforms is low [39]. Several studies have also compared the use of microarray and real-time RT-PCR for establishing miRNA profiles and have reported that the actual overlap between differentially expressed miRNAs was low when microarray and real-time RT-PCR data were compared [40], [41]. Multiple factors could contribute to this low interplatform consistency, such as data collection, mining, and noise subtraction [39]. Further methodological studies are required to improve interplatform reproducibility and address this limitation of miRNA studies.

## References

[1] Stefani G, Slack FJ (2008) Small non-coding RNAs in animal development. Nat Rev Mol Cell Biol 9: 219–230. 18270516 10.1038/nrm2347

[2] Ambros V (2004) The functions of animal microRNAs. Nature 431: 350–355. 15372042 10.1038/nature02871

[3] Bartel DP (2004) MicroRNAs: genomics, biogenesis, mechanism, and function. Cell 116: 281–297. 14744438 10.1016/s0092-8674(04)00045-5

[4] Friedman RC, Farh KK, Burge CB, Bartel DP (2009) Most mammalian mRNAs are conserved targets of microRNAs. Genome Res 19: 92–105. 18955434 10.1101/gr.082701.108 PMC2612969

[5] Calin GA, Ferracin M, Cimmino A, Di Leva G, Shimizu M, et al (2005) A MicroRNA signature associated with prognosis and progression in chronic lymphocytic leukemia. N Engl J Med 353: 1793–1801. 16251535 10.1056/NEJMoa050995

[6] Xu P, Vernooy SY, Guo M, Hay BA (2003) The Drosophila microRNA Mir-14 suppresses cell death and is required for normal fat metabolism. Curr Biol 13: 790–795. 12725740 10.1016/s0960-9822(03)00250-1

[7] Lawrie CH, Gal S, Dunlop HM, Pushkaran B, Liggins AP, et al (2008) Detection of elevated levels of tumour-associated microRNAs in serum of patients with diffuse large B-cell lymphoma. Br J Haematol 141: 672–675. 18318758 10.1111/j.1365-2141.2008.07077.x

[8] Valadi H, Ekstrom K, Bossios A, Sjostrand M, Lee JJ, et al (2007) Exosome-mediated transfer of mRNAs and microRNAs is a novel mechanism of genetic exchange between cells. Nat Cell Biol 9: 654–659. 17486113 10.1038/ncb1596

[9] Arroyo JD, Chevillet JR, Kroh EM, Ruf IK, Pritchard CC, et al (2011) Argonaute2 complexes carry a population of circulating microRNAs independent of vesicles in human plasma. Proc Natl Acad Sci U S A 108: 5003–5008. 21383194 10.1073/pnas.1019055108 PMC3064324

[10] Kosaka N, Iguchi H, Yoshioka Y, Takeshita F, Matsuki Y, et al (2010) Secretory mechanisms and intercellular transfer of microRNAs in living cells. J Biol Chem 285: 17442–17452. 20353945 10.1074/jbc.M110.107821 PMC2878508

[11] Pegtel DM, Cosmopoulos K, Thorley-Lawson DA, van Eijndhoven MA, Hopmans ES, et al (2010) Functional delivery of viral miRNAs via exosomes. Proc Natl Acad Sci U S A 107: 6328–6333. 20304794 10.1073/pnas.0914843107 PMC2851954

[12] Wang K, Zhang S, Weber J, Baxter D, Galas DJ (2010) Export of microRNAs and microRNA-protective protein by mammalian cells. Nucleic Acids Res 38: 7248–7259. 20615901 10.1093/nar/gkq601 PMC2978372

[13] Moritani T, deVries HA (1979) Neural factors versus hypertrophy in the time course of muscle strength gain. Am J Phys Med 58: 115–130. 453338

[14] Staron RS, Karapondo DL, Kraemer WJ, Fry AC, Gordon SE, et al (1994) Skeletal muscle adaptations during early phase of heavy-resistance training in men and women. J Appl Physiol 76: 1247–1255. 8005869 10.1152/jappl.1994.76.3.1247

[15] Consitt LA, Copeland JL, Tremblay MS (2002) Endogenous anabolic hormone responses to endurance versus resistance exercise and training in women. Sports Med 32: 1–22. 11772159 10.2165/00007256-200232010-00001

[16] Hakkinen K, Pakarinen A (1993) Acute hormonal responses to two different fatiguing heavy-resistance protocols in male athletes. J Appl Physiol 74: 882–887. 8458810 10.1152/jappl.1993.74.2.882

[17] Hakkinen K, Pakarinen A, Newton RU, Kraemer WJ (1998) Acute hormone responses to heavy resistance lower and upper extremity exercise in young versus old men. Eur J Appl Physiol Occup Physiol 77: 312–319. 9562359 10.1007/s004210050339

[18] Kraemer WJ, Ratamess NA (2005) Hormonal responses and adaptations to resistance exercise and training. Sports Med 35: 339–361. 15831061 10.2165/00007256-200535040-00004

[19] Mitchell PS, Parkin RK, Kroh EM, Fritz BR, Wyman SK, et al (2008) Circulating microRNAs as stable blood-based markers for cancer detection. Proc Natl Acad Sci U S A 105: 10513–10518. 18663219 10.1073/pnas.0804549105 PMC2492472

[20] Baggish AL, Hale A, Weiner RB, Lewis GD, Systrom D, et al (2011) Dynamic regulation of circulating microRNA during acute exhaustive exercise and sustained aerobic exercise training. J Physiol 589: 3983–3994. 21690193 10.1113/jphysiol.2011.213363 PMC3179997

[21] Recommendations of the German Society for Clinical Chemistry (1977) Standard method for the determination of creatine kinase activity. J Clin Chem Clin Biochem 15: 255–260. 16981

[22] Wada S, Kato Y, Okutsu M, Miyaki S, Suzuki K, et al (2011) Translational suppression of atrophic regulators by microRNA-23a integrates resistance to skeletal muscle atrophy. J Biol Chem 286: 38456–38465. 21926429 10.1074/jbc.M111.271270 PMC3207415

[23] Costill DL, Fink WJ (1974) Plasma volume changes following exercise and thermal dehydration. J Appl Physiol 37: 521–525. 4415099 10.1152/jappl.1974.37.4.521

[24] Dill DB, Costill DL (1974) Calculation of percentage changes in volumes of blood, plasma, and red cells in dehydration. J Appl Physiol 37: 247–248. 4850854 10.1152/jappl.1974.37.2.247

[25] Jensen SG, Lamy P, Rasmussen MH, Ostenfeld MS, Dyrskjot L, et al (2011) Evaluation of two commercial global miRNA expression profiling platforms for detection of less abundant miRNAs. BMC Genomics 12: 435. 21867561 10.1186/1471-2164-12-435 PMC3184117

[26] Tamayo P, Slonim D, Mesirov J, Zhu Q, Kitareewan S, et al (1999) Interpreting patterns of gene expression with self-organizing maps: methods and application to hematopoietic differentiation. Proc Natl Acad Sci U S A 96: 2907–2912. 10077610 10.1073/pnas.96.6.2907 PMC15868

[27] Drummond MJ, McCarthy JJ, Fry CS, Esser KA, Rasmussen BB (2008) Aging differentially affects human skeletal muscle microRNA expression at rest and after an anabolic stimulus of resistance exercise and essential amino acids. Am J Physiol Endocrinol Metab 295: E1333–1340. 18827171 10.1152/ajpendo.90562.2008 PMC2603551

[28] Nielsen S, Scheele C, Yfanti C, Akerstrom T, Nielsen AR, et al (2010) Muscle specific microRNAs are regulated by endurance exercise in human skeletal muscle. J Physiol 588: 4029–4037. 20724368 10.1113/jphysiol.2010.189860 PMC3000590

[29] Uhlemann M, Mobius-Winkler S, Fikenzer S, Adam J, Redlich M, et al.. (2012) Circulating microRNA-126 increases after different forms of endurance exercise in healthy adults. Eur J Prev Cardiol. In press. 10.1177/2047487312467902 23150891

[30] Wang JF, Yu ML, Yu G, Bian JJ, Deng XM, et al (2010) Serum miR-146a and miR-223 as potential new biomarkers for sepsis. Biochem Biophys Res Commun 394: 184–188. 20188071 10.1016/j.bbrc.2010.02.145

[31] Wang G, Tam LS, Li EK, Kwan BC, Chow KM, et al (2010) Serum and urinary cell-free MiR-146a and MiR-155 in patients with systemic lupus erythematosus. J Rheumatol 37: 2516–2522. 20952466 10.3899/jrheum.100308

[32] Gui J, Tian Y, Wen X, Zhang W, Zhang P, et al (2011) Serum microRNA characterization identifies miR-885-5p as a potential marker for detecting liver pathologies. Clin Sci (Lond) 120: 183–193. 20815808 10.1042/CS20100297 PMC2990200

[33] Bala S, Tilahun Y, Taha O, Alao H, Kodys K, et al (2012) Increased microRNA-155 expression in the serum and peripheral monocytes in chronic HCV infection. J Transl Med 10: 151. 22846613 10.1186/1479-5876-10-151 PMC3477071

[34] Oerlemans MI, Mosterd A, Dekker MS, de Vrey EA, van Mil A, et al.. (2012) Early assessment of acute coronary syndromes in the emergency department: the potential diagnostic value of circulating microRNAs. EMBO Mol Med. 10.1002/emmm.201201749 PMC3494874 23023917

[35] Kawaguchi T, Komatsu S, Ichikawa D, Morimura R, Tsujiura M, et al (2013) Clinical impact of circulating miR-221 in plasma of patients with pancreatic cancer. Br J Cancer 108: 361–369. 23329235 10.1038/bjc.2012.546 PMC3566805

[36] Pu XX, Huang GL, Guo HQ, Guo CC, Li H, et al (2010) Circulating miR-221 directly amplified from plasma is a potential diagnostic and prognostic marker of colorectal cancer and is correlated with p53 expression. J Gastroenterol Hepatol 25: 1674–1680. 20880178 10.1111/j.1440-1746.2010.06417.x

[37] Song MY, Pan KF, Su HJ, Zhang L, Ma JL, et al (2012) Identification of serum microRNAs as novel non-invasive biomarkers for early detection of gastric cancer. PLoS One 7: e33608. 22432036 10.1371/journal.pone.0033608 PMC3303856

[38] Li J, Wang Y, Yu W, Chen J, Luo J (2011) Expression of serum miR-221 in human hepatocellular carcinoma and its prognostic significance. Biochem Biophys Res Commun 406: 70–73. 21295551 10.1016/j.bbrc.2011.01.111

[39] Wang B, Howel P, Bruheim S, Ju J, Owen LB, et al (2011) Systematic evaluation of three microRNA profiling platforms: microarray, beads array, and quantitative real-time PCR array. PLoS One 6: e17167. 21347261 10.1371/journal.pone.0017167 PMC3037970

[40] Git A, Dvinge H, Salmon-Divon M, Osborne M, Kutter C, et al (2010) Systematic comparison of microarray profiling, real-time PCR, and next-generation sequencing technologies for measuring differential microRNA expression. RNA 16: 991–1006. 20360395 10.1261/rna.1947110 PMC2856892

[41] Sato F, Tsuchiya S, Terasawa K, Tsujimoto G (2009) Intra-platform repeatability and inter-platform comparability of microRNA microarray technology. PLoS One 4: e5540. 19436744 10.1371/journal.pone.0005540 PMC2677665

